# Genuine Movement Learning Through a Deleuzian Approach

**DOI:** 10.3389/fspor.2021.771101

**Published:** 2021-12-07

**Authors:** Håkan Larsson, Gunn Nyberg, Dean Barker

**Affiliations:** ^1^Department of Movement, Culture and Society, The Swedish School of Sport and Health Sciences, Stockholm, Sweden; ^2^Department of Teacher Education, Dalarna University, Falun, Sweden; ^3^Department of Health Sciences, Örebro University, Örebro, Sweden

**Keywords:** movement learning, Deleuze, genuine learning, juggling, unicycling, dancing, teaching

## Abstract

The purpose of the article is to outline how Deleuzian concepts, notably the notions of apprenticeship in signs based on a pedagogy of the concept, can stimulate thinking and understanding of movement learning, and provide insights about pedagogical implications in various movement educational settings. Methodologically, the article falls somewhere in between theoretical exposition and presentation of original empirical research, i.e., a “theoreticoempirical” exposition. We borrowed some ideas formulated by Deleuze (and Guattari), which have been further developed by educational researchers, about “an apprenticeship in signs” based on “a pedagogy of the concept,” to analyse situations where students explore new movements. We use material generated from pedagogical interventions comprising of exploration of kinescapes. In these interventions, school and university students are encouraged to explore, and learn, juggling, unicycling and dancing. Findings indicate how students pass through interpretative illusions until some of them grasp difference in itself in what could be called its immanent differentiation of the actual, i.e., they learn how to juggle, unicycle or dance. This is what we designate *genuine learning*. The triadic relation between concepts, percepts and affects offer us clues to what juggling, unicycling or dancing mean to learners (concepts), what learners pay attention to while practising (percepts), and what gets them moving (affects). Importantly, through viewing learning as an apprenticeship in signs, the Deleuzian approach reminds us that the triadic relation is open-ended, meaning that concepts, percepts and affects are never final but always a potential actualisation. Concepts, percepts and affects are constantly in the process of becoming. Since genuine learning is not about narrowing down how a movement should be executed and experienced, the task of a movement educator could, then, be to accompany learners in explorative pursuits. In this way, teachers can help learners escape preconceptions about movements (who can do what and when) and instead explore new movement opportunities.

## Introduction

This paper is has sprung from our amazement about the variation regarding how a number of secondary school students and university students approached our invitation to explore—and learn—new movements.

In a secondary school gym, a class of students aged 15–16 years are invited to practise juggling. It is clear among the students and the teacher, as well as the researchers, that what it means to “juggle” is basically to manage to juggle with at least three balls in a cascade pattern (starting with one ball in each hand and throwing the balls to the opposite hand, but with a little delay, so that the pattern becomes throw-throw-catch-catch). The students have 10 lessons available to develop their juggling capabilities. A few students seem already at the outset to be able to juggle using the cascade pattern, but most of the students cannot. While some of the beginners manage to juggle cascade style already during the first lesson, others struggle initially, and learn to juggle after some lessons. Still other students appear neither able to—nor interested in—learning to juggle at the end of the ten lesson unit—at least not on the basis of the existing pedagogical arrangements.

In a gym hall located at a university, a group of teacher education students are invited to practise unicycling. Perhaps even stronger than in the juggling context, it is very clear what this capability means among students, teacher educator and researchers. Two students are able to ride a unicycle already at the outset of the unicycling unit, but most of them find even mounting the cycle with support from a wall or a railing to be challenging. Nevertheless, after two two-h practise sessions, some students manage to unicycle 40 m across the gym. After the whole five-session unit, more students are able to do the same, but there are also a number of students who still find it challenging to even mount the unicycle, let alone pedal some metres across the floor. A few of students have also dropped out of the practise.

In another university gym hall, a group of sports coaching students are invited to practise dance moves and compose a routine in groups. In this case, there are certainly strong norms regarding the meaning of dance ability and who can and cannot dance. Nevertheless, it is clear that there is a difference with both juggling and unicycling in the sense that to some students it is less clear what dancing ability means and whether they can dance. Moreover, the students' approaches to practising are varied to say the least. Some want to try right away. Others want to observe the teacher or a YouTube clip carefully and practise only after a while. Some students proceed immediately to the “whole” dance movement, while others prefer to divide the movement into “constitutive parts” that they (and to some extent their teacher and the researchers) seem to believe make up the “whole” dance movement. After five two-h practise sessions, small groups of students perform various short dance routines that they have put together.

All the above occasions occurred during the implementation of a research project that has the aim of theorising movement learning in new ways (see e.g., Barker et al., 2021; Nyberg et al., 2021). Questions about movement learning have indeed become topical within physical education and sport pedagogy research lately (Larsson, 2021; *Physical Education and Sport Pedagogy*, p. 26:3), where, summarily, so-called linear approaches have been challenged by non-linear approaches (e.g., Renshaw et al., 2010; Davids et al., 2012; Chow et al., 2015). We will return to linear and non-linear approaches to movement learning in a while, but first some things must be said about why we believe that developing new understandings of movement learning is important and what our purpose is with this particular article.

Why are we interested in movement learning? And why are we interested in developing new understandings of movement learning? Such questions are always difficult to answer exhaustively, but at least partly, we believe that it has become virtually impossible to discuss movement learning in the school subject physical education (Nyberg and Larsson, 2014; Larsson and Nyberg, 2017), perhaps especially in Scandinavian countries. We agree with Evans (Evans, 2004, p. 96) when he suggests that physical education as a school subject is becoming “strangely disembodied.” In a Swedish context, as elsewhere, this means that propositional knowledge is considered more and more important (i.e., knowledge *about* health issues), and that “movement learning” is taken for granted to mean “learning of pre-defined sports skills” (and is thus not seen as suitable content in a health-oriented subject). In relation to Arnold's (Arnold, 1979) classic distinction between education in, through and about movement, there seems to be less and less focus on education *in* movement, chiefly because “education in movement” (just as with “movement learning”) is interpreted narrowly. This development echoes, we believe, Herold and Waring's (Herold and Waring, 2017) question “Is practical subject matter knowledge still important [in physical education teacher education]?”

Our aspiration to formulate new perspectives of movement learning stems mainly from our engagement with school physical education and teacher education in this subject. We believe nonetheless, that developing new perspectives of movement learning could be fruitful also in other movement culture domains, such as in competitive sports and fitness, two areas where equally “square” approaches to movement learning dominate, and where pedagogical approaches are similarly connected to strong societal norms regarding, for example, age, gender, socioeconomics, ethnicity, ability, and so on. These norms may in turn contribute to individuals' ceasing to learn new ways of moving at an early age as well as marginalisation and exclusion of people, bodies and movements that are not considered perfect (Barker et al., 2021). This does not mean that we are attempting to offer *the* answer to how to approach movement learning. Rather than providing an indisputable answer, we contend that it is more important to explore alternatives, and to seek out new approaches to learning, approaches that have been developed elsewhere, and that can contribute to the issue of movement learning.

In our endeavour to find alternative approaches to movement learning, we seek inspiration from the French philosopher Gilles Deleuze (1925–1995). We are curious about what Deleuze has to offer regarding the issue of movement learning because he seems to have sparked inspiring new understandings of physical activity (Markula, 2019) and physical education (Landi, 2019), as well as of education and learning more generally (Semetsky, 2008; Cole, 2015). Moreover, we were intrigued by what Deleuze himself has said about learning:

“Learning is the appropriate name for the subjective acts carried out when one is confronted with the objecticity of a problem (Idea) […] to learn is to enter into the universal of the relation which constitute the Idea, and into their corresponding singularities […] We never know in advance how someone will learn: by means of what loves someone becomes good at Latin, what encounters make them a philosopher, or in what dictionaries they learn to think” (Deleuze, 1994, p. 164f).

While these sentences may in a sense be enigmatic, we have a feeling that they might point us in directions where movement learning can be understood in radically new ways. In particular, we have been caught by what Semetsky (2008) boldly calls “genuine learning,” that is, the learning that “proceeds through a deregulation of the senses and a shock that compels thought against its will to go beyond its ordinary operations” (p. x); a learning that takes us beyond ““stock notions,” “natural” or “habitual” modes of comprehending reality” (Bogue, 2004, p. 328, with reference to Deleuze, 2000, p. 4). To clarify the notion of genuine learning, we have found inspiration in Bogue's (Bogue, 2004) exposition on what he calls *apprenticeship in signs*, which constitutes “the accession to a new way of perceiving and understanding the world” (p. 328). Further, we draw on the notion of *a pedagogy of the concept*, which according to Semetsky (2015, p. 4) is about “practical learning from experience oriented to real-life problems that defy univocal solutions but represent experimentation with the world and ourselves. Concepts are invented in practise and cannot be reduced to any a priori theoretical judgment.” In this article, we will use the notions of an apprenticeship in signs based on a pedagogy of the concept to designate genuine learning.

The purpose of the article is to outline how Deleuzian concepts, notably the notions of apprenticeship in signs based on a pedagogy of the concept, can stimulate thinking and understanding of movement learning, and provide insights about pedagogical implications in various movement educational settings. Since we are primarily empirical educational researchers, not philosophers, and since Deleuze's writings are often recognised as somewhat difficult to access (Stanford Encyclopaedia of Philosophy, Gilles Deleuze), we will enlist the help of Deleuze interpreters within education, primarily Bogue (2004) and Semetsky (2008, 2009) and, in our efforts to develop a new perspective of movement learning. Moreover, since Deleuze himself (Deleuze, 1994) used a movement activity to exemplify learning—swimming, an example that is further unfolded by Bogue (2004), we will extend our account from swimming to a number of empirical examples from our own research, namely juggling, unicycling and dancing. However, before we move on to considering genuine learning, we believe there is a need to at least briefly relate to current approaches to movement learning, which are generally referred to as linear or non-linear approaches.

## Linear and Non-linear Approaches to Movement Learning

Approaches to movement learning have often relied on a linear logic. According to this logic, all learners progress in similar ways from not having a particular capability (or skill), to having the capability in a basic or immature form, to having the capability in a complex, advanced form (Coker, 2018). A cognitive transition is expected to accompany physiological and biomechanical changes, and learners are expected to advance from slow, conscious control of one's body to an automated performance (Beilock and Carr, 2001). In a developmental sense, the final objective of linear learning is to be able to perform ideal movement patterns without simultaneously attending to those movements.

Linear logic of movement learning is evident in research, policy and practise. A number of research investigations involve pre- and post-tests and aim to measure individuals' improvements over a given time period (Chen et al., 2016; Bedard et al., 2020). Physical education curricula too, frequently organise learning objectives in progressive steps. Pupils are expected to develop basic movement capabilities in approximate accordance with age and developmental maturity (Brian et al., 2017) and gradually learn to adapt those basic capabilities in complex situations (Janemalm et al., 2020). In physical education lessons, linear logic exists in drill- and technique-oriented practises. A linear logic supports teacher-centred pedagogies characterised by demonstrations of an ideal performance and opportunities for formal practise. Chen et al. (2017) exemplify the linear logic in physical education practise when they suggest that “children's motor skill development occurs best when children learn and practise the skill through engaging in sequential learning tasks within structured learning environments based on children's sequence of motor skill development” (p. 223).

Since the 1980s, researchers have been concerned to offer alternatives to the linear perspective (Sheets-Johnstone, 2011; Varela et al., 2017). Probably the most investigated alternatives in PE scholarship fall under the heading of constraints-led approaches (Renshaw et al., 2016; Renshaw and Chow, 2019). Constraints-led approaches are based on ecological dynamics and are closely related to non-linear pedagogy (Chow et al., 2006). The main tenets of constraints-led approaches are that: (i) learners, tasks and environments are seen to have unique characteristics that constrain and afford certain actions; (ii) these characteristics interact with one another making a person's actions emergent rather than linear or predictable; (iii) new situations make novel demands of learners and therefore require some form of adaptation, and (iv) learners perceive opportunities for action within their environment so perception allows for action. At the same time, acting (or moving) creates new opportunities for perception (Correia et al., 2019; Roberts et al., 2019). In this sense, action and perception are “coupled.” Together, these principles suggest that learning is too multifaceted, too individualised, and too context-dependent to be adequately captured by linear thinking.

Constraints-led approaches have not been the only alternatives to non-linear logic. Some perspectives on children's movement learning are based on the idea that learning does not take place in linear ways and that movement capability should be fostered through playful activity (Baumgarten, 2006). Many game sense approaches also foreground play and involve a non-linear logic (Harvey and Jarrett, 2014). Games sense approaches typically assume that tactical understanding and technical competence develop concomitantly, and therefore challenge the linear idea of first learning basic techniques and then progressing to complex game situations (Harvey et al., 2018). Our own approach to movement learning, which we call kinesiocultural exploration and which will be further outlined below, can also be considered non-linear (Barker et al., 2021; Nyberg et al., 2021). In this work, our broad goal has been to develop an alternative theoretical perspective to the mechanistic way of thinking that has influenced much movement learning research. We have proposed that movement capability involves an appreciation and embodied sensitivity to biomechanical, experiential and sociocultural movement factors and that this develops through processes of experimentation and exploration of kinescapes (movement landscapes) rather than through direct instruction and drill.

## Genuine Learning

Deleuze's writings, including his work with Guattari, are extensive, and although he only paid marginal attention to questions about education, teaching and learning, he repeatedly touched upon such issues in significant ways (see, e.g., Peters, 2004; Semetsky, 2008; Cole, 2015). It is challenging to capture Deleuze's overall “project” in a nutshell, but broadly, he was concerned with philosophical matters. According to one encyclopaedia (Britannica, Gilles Deleuze), Deleuze's writings can be divided into two periods. In a first period, his main ambition was to challenge the dominance in Western philosophy of unity over multiplicity, and of sameness over difference. In a second period, together with Guattari, he wanted to free psychoanalysis from normalisation and control. Both unity and sameness, and normalisation and control can be said to dominate linear approaches to movement learning, and possibly also non-linear approaches. In a way, Deleuze and Guattari's attack on unity and sameness, and normalisation and control are included in what they called a pedagogy of the concept (Deleuze and Guattari, 1994).

It is not easy, based on Deleuze and Guattari's own account, to determine what the expression pedagogy of the concept means, particularly since their ethos was not to “narrow down” meaning, but instead to “open up.” Deleuze and Guattari (1994) asked for instance, “What's the best way to follow the great philosophers? Is it to repeat what they said or to do what they did, that is, create concepts for problems that necessarily change?” (p. 28). Consequently, they point to how the performative function of concepts, what concepts *do*, can be pursued, rather than their representative function, what they mean: “[I]n philosophy, concepts are only created as a function of problems which are thought to be badly understood or badly posed (pedagogy of the concept)” (Deleuze and Guattari, 1994, p. 16). This way of putting it can, we believe, point to how movement learning can be understood, and how learners approach challenging movement tasks. Thus, Deleuzian thinking dissolves the distinction between observer and practitioner, much in the same way as Deleuze dissolves the distinction between teacher and student/learner (Deleuze, 2000; see also Bogue, 2004). We will return to the issue of teaching in the discussion.

In a series of texts, educational researcher Semetsky (2008, 2009) unfolds further the notion of a pedagogy of the concept in ways that have been fruitful for our understanding of movement learning. She maintains that a pedagogy of the concept posits “a triadic relation encompassing percepts, affects, and concepts, so that novel concepts [can] be invented or created as a function of real experience” (Semetsky, 2009, p. 443). Importantly, we “need all three to *get things moving*” (Deleuze, 1995, p. 165, cited in Semetsky, 2009, p. 449; original emphasis). Since Deleuze was a philosopher, it is unlikely that he was thinking about juggling or dancing when he referred to moving. However, since Deleuze's approach was explicitly anti-dualist, we take it that moving could designate both intellectual movement and bodily movement, which do not necessarily have to be divided. The aspect of moving is key also in the sense that, to Deleuze, nothing is fixed. Reality is for Deleuze, always in the process of *becoming* (Deleuze and Guattari, 2013). This idea connects to his focus of multiplicity and difference. Emphasising the triadic relation between percepts, affects and concepts does not mean, then, that this relation should be fixed—“then you get it.” Rather, the triad is in constant flux, which means that the constitutive parts change in tandem; and one way of conceptualising and perceiving some “thing” is always temporary or provisional. In short, genuine learning is about *becoming-other* (Semetsky, 2009), and there is no fixed starting or ending to the learning process. On the contrary, similar to what Aggerholm (2021) writes about practising, we believe that learning is open-ended. “Being able to perform a specific movement opens up for new movements and refinements” (Aggerholm, 2021, p. 130).

As researchers of movement learning, we take it that when Deleuze writes about “concepts” and “understanding,” in an anti-dualist vein he does not necessarily limit the meaning of these terms to abstract concepts and cognitive understanding. Thus, concepts and understanding are experienced rather than thought. That is, what some*thing* (such as a movement, or a sequence of movements) “is,” or means, is bodily experienced/perceived/discerned. Now we are moving towards another aspect of the triad, percepts, which designates what is perceived; or to use Polanyi's (Polanyi, 2012) expression, what is in somebody's focal or subsidiary attention. A percept, according to Semetsky (2009, p. 448), “is a perception in becoming; therefore, it means perceiving something that is not given.” Rather, what is perceived is always a potential “something”; never a fixed state of being, but always in the process of becoming. Affects, the third aspect of the triad, can be seen as the driving force of the equation. According to the translator of Deleuze and Guattari's (Deleuze and Guattari, 2013) book *A Thousand Plateaus*, affect “is a pre-personal intensity corresponding to the passage from one experiential state of the body to another and implying an augmentation or diminution in that body's capacity to act” (p. xv; Notes on the Translation and Acknowledgements). Thus, what affects a body's capability is neither external/objective nor internal/subjective but emerges through a learner's engagement with a practise. Remember what Deleuze wrote about learning: it is “the appropriate name for the subjective acts carried out when one is confronted with the objecticity of a problem (Idea)” (Deleuze, 1994, p. 164; we believe that the term “objecticity” is a play with words, a hybrid between “objectivity” and “authenticity”). However, being confronted with the objecticity of a problem is not tantamount to getting into direct contact with the problem. Rather, it is about what Deleuze (2000) called an apprenticeship in signs.

The theme of apprenticeship in signs appears in a text called *Proust and Signs* (Deleuze, 2000), where Deleuze delved into the literary work of author Marcel Proust. “Everything that teaches us something,” Deleuze contends, “emits signs; every act of learning is an interpretation of signs or hieroglyphs. The term “sign” needs to be somewhat problematised. Signs *mean* things, that is, they have representational value. However, in Deleuzian and other poststructuralist theory signs also *do* things, that is, they are performative. Movement actions, for example, are performed at the same time as they mean something. Importantly, in poststructuralist theory, signs never have a fixed meaning. The meaning of a sign/movement, then, is always in-becoming, which is significant for the implication of an apprenticeship in signs. Interestingly, in French, apprenticeship is translated to *apprentissage*, which is related to the verb “to learn”: *apprendre*. It is worth noticing that Deleuze wrote “every*thing* that teaches us,” indicating that people learn in all kinds of situations, and not only from a teacher. In *Difference and Repetition*, for example, Deleuze (1994, p. 23) assigns the swimming teacher a rather limited role, for “the movements of the swimming instructor which we reproduce on the sand bear no relation to the movements of the wave, which we learn to deal with only by grasping the former in practise as signs.” According to Bogue (2004, p. 341), “[t]o learn is to encounter signs, to undergo the disorienting jolt of something new, different, truly other, and then to explicate those signs, to unfold the differences they enfold.”

Learning new movements, then, is far from “programming” new movements, as is often assumed in linear sports practises. Rather, genuinely learning new movements is about solving movement problems through attending to, and grasping, movement signs that the practise emits. As one does so, Bogue (2004, p. 341) continues,

one passes through objective and subjective interpretative illusions until one grasps difference itself in its immanent differentiation within the actual. […] [O]ne sees as well the virtual domain of difference in itself, which is not an amorphous chaos, but an infinite collection of structured problems. Each problem consists of a general set of differentially related elements and their corresponding singular points, or zones of potential actualization. Genuine learning involves an engagement with such problems, a re-orientation of thought following its initial disorientation, such that thought may comprehend something new in its newness, as a structured field of potential metamorphic forces rather than a pre-formed body of knowledge to be mastered.

It is not difficult, we believe, to imagine juggling, unicycling and dancing learning as passages through “interpretive illusions.” Involving the triadic relation between concepts, percepts and affects, learning juggling for example, can be seen as a re-orientation of the preliminary concept one has of what juggling “is” in a manner that new concepts can be formed, and which materialise in conjunction with that the learner perceives she or he is doing, and manages to pay attention to in the movement practise. There remains however, the question of what affects genuine learning into becoming. We will soon explore this question in our empirical examples.

## Kinesiocultural Exploration as a Way of Examining Genuine Learning

This article falls somewhere in between theoretical exposition and a presentation of original empirical research and can be termed a “theoreticoempirical” exposition. However, since we will display a number of empirical examples of what we see as genuine learning, as well as situations where the intended learning apparently never happened, we offer a brief account of how our research was conducted.

Developing new ways of approaching/understanding/theorising movement learning was the primary purpose of the MOVE research project. At the outset, we were highly inspired by Ryle's problematisation of “mind” (Ryle, 2009) and Polanyi's notion of “personal knowledge” (Polanyi, 2012). Since these thinkers emphasise exploration and attention, we invited participants in different movement learning contexts to explore movements and movement practises. The movement practises: juggling, unicycling and dancing, were selected mainly because they are not mainstream sports. Moreover, at least with juggling and unicycling, we anticipated that it would be fairly obvious to the learners when they “knew it.” The participants were secondary school students (juggling) and university students (unicycling and dancing) who all volunteered, together with their respective teachers, to take part in the research project. Juggling was practised during physical education lessons (three classes for 8–10 lessons à 45 min); dancing was practised during a university course (about movement learning; two groups for five two-h lessons); while unicycling was practised during specially arranged sessions (two groups for five two-h sessions). To get both an observer and participant perspective, we documented the lessons/sessions using field-notes, GoPro-cameras and student logbooks. The GoPro-cameras were used for “filming-at-a-distance” as well as capturing conversations between students and between students, teachers and researchers.

The general approach to movement exploration was designed by us, but the concrete planning was done in collaboration between us and the respective groups' teachers. Overall, we attempted to avoid linear designs based on direct instruction and particular sequencing. Together with the teachers, we “staged the scene” for exploration in the sense that we offered a variety of tasks and equipment with which the students could explore educational movement landscapes, or *kinescapes* (Nyberg et al., 2020, 2021). Kinescapes are movement (in Greek, *kinesis*) landscapes, which “have their own features and principles that relate to propulsion, flight, rotation and so forth” (Barker et al., 2021, p. 7). Further, Barker et al. (2021, p. 7–8) point out that:

[p]rinciples are not only mechanical but also cultural and aesthetic as they encompass traditions and expectations relating to good performance (c.f. Shusterman, 2012). A cartwheel performed in the kinescape of artistic gymnastics, for example, constitutes a qualitatively different movement experience to a cartwheel performed during a capoeira routine. (Barker et al., 2021, p. 7–8).

The non-linear approach does not mean, however, that we entirely avoided making suggestions to the participants about what they could do or how they could approach a challenging movement task. It should be emphasised, though, that we are not experts of any of these movement practises. We offered comments and suggestions as best we could, often in cases where students approached us with questions or requests for feedback. At times, we also referred participants to YouTube clips of juggling, unicycling or dancing, that were aimed at expert, average and beginner practitioners.

Although we were not knowledgeable about Deleuze's thinking about learning at the outset of the project, we believe that his overall *geophilosphical* method (Deleuze and Guattari, 2013; see also Peters, 2004) resonates relatively well with our notion of kinescapes and *kinesiocultural exploration* (Barker et al., 2021; Larsson et al., 2021). Kinesiocultural exploration designates the embodied exploration of kinescapes, where learners gradually discover the critical cultural, material, and mechanical elements that are necessary to master/perform/participate—and possibly also change/adapt—in a movement practise. This is where we believe that kinesiocultural exploration can add to the existing literature, which rarely addresses multiple aspects such as cultural, material and mechanical elements. Additionally, the kinesiocultural exploration approach emphasised the element of attention, that is, the ability to discern critical elements of the movement practise, as well as the ability to judge, or weigh, to use a more bodily expression, critical features of the kinescape to allow for appropriate action. This is where we believe that Deleuzian thinking ties in quite nicely into our work. At the same time, it has also challenged us, and provoked new lines of flight in our thinking (cf. Deleuze and Guattari, 2013). These are issues that we will now draw attention to through some empirical examples.

## Some Empirical Examples

Now, we will offer a number of examples from our extensive empirical material, where we believe that the notions of an apprenticeship in signs based on a pedagogy of the concept have been particularly fruitful for our understanding of movement learning.

### Juggling

It is the first lesson for one of the secondary school classes who participated in practising juggling. After an “initial warm-up,” where the teacher invited the students to throw and catch various objects (balls, skittles, scarves, rings, etc.), the students are requested to choose objects and start exploring juggling in pairs. One of the researchers approaches two girls, Adele and Beth, who are trying to juggle with two balls. They move the balls in a circular pattern, where they throw the balls from the right hand to the left on a high plane, and then toss the ball from the left hand to the right on a lower plane. The researcher invites Adele and Beth to read the assignment which is written on a piece of paper lying in front of them on the floor (which asks its readers to ponder how it would be possible to proceed from juggling with two balls to juggling with three balls). The girls hesitate. After a while, the researcher asks: “What happens if you add a ball? … What would you do with it?” Adele stops. She seems to be thinking/feeling, and then she tries to throw the balls in a cascade pattern. “That doesn't work!” she cries out. The researcher does not pay any attention to the remark, but asks instead: “Did you notice what you did there?” “Yes,” she responds. “So, the crux is to throw one and then the other,” the researcher adds. Adele mimics the movement pattern, but without actually throwing the balls. After a little more time to “think-feel” she throws the balls in the cascade pattern but pauses in between each sequence of throw-throw-catch-catch. She still seems unsure. Now the teacher approaches the girls and the researcher. Adele asks the teacher: “Hey, [teacher's name], can you show me?” The teacher explains the cascade pattern. “This is the first step to be able to proceed with juggling with several balls.” Adele tries again and cries out: “Is this right, then?” She then goes back and forth between the circular pattern and the cascade pattern, as if comparing the two patterns. After another couple of minutes, Adele tries with three balls and immediately succeeds with one sequence of the cascade pattern. She looks at the researcher with a surprised face. “I haven't been able to do this before!” She then continues to practise juggling the cascade pattern.

To us, it seems as if Adele starts off with an initial concept of juggling as “throwing-balls-in-a-circular-pattern.” The researcher's question about what will happen if she would throw a third ball constitutes a deregulation of the senses that, eventually, compels her to move beyond her ordinary operations (affect). Initially, when Adele explores the cascade pattern, she seems to be uncertain about both how to interpret the signs that her attempts to throw in a cascade pattern emits (percept) and whether what she is doing is actually “right” (concept). The teacher's involvement seems to convince her that she *is* on to something (affect). She then seems to compare the cascade pattern with the circular pattern in order to clarify to herself what she should pay attention to when going further with practising the cascade pattern (percept). The joy and satisfaction marked by her surprised face and the comment that she has not been able to do this before designates a confirmation that she has made a qualitative change regarding what juggling “is” (concept) and what she must pay attention to when she continues practising juggling (percept). This confirmation, however, does not mark the end of her learning process. Quite to the contrary, it compels Adele, and Beth, to continue practising (affect), and to explore juggling in new ways and with new material (skittles, rings, bigger balls, etc.).

In another publication (Barker et al., 2020), situations like the above one are theorised as “learning thresholds,” which we now understand as another way of describing genuine learning, i.e., a qualitatively changed way of perceiving and acting in the world. It should be noted, however, that students reached these thresholds in different ways. Thus, while the above example illustrates relationships between concepts, affects and percepts, it shows one way that students approached and learned (how) to juggle. Moreover, not all students managed to learn to juggle, if “juggling” refers to the ability to throw and catch more than two balls in a cascade pattern. In Larsson et al. (2021), for example, we suggest that gender norms affect how students conceive juggling and what they pay attention to while practising. That study indicates that gender norms seem to encourage many boys to juggle in a creative fashion and explore ever differing ways of throwing and catching. In contrast, girls seem to be encouraged to refine a certain juggling technique through persistent practise. However, there are always exceptions to these “rules.”

### Unicycling

A group of some 25 teacher students have volunteered to practise unicycling. Most of them have not even attempted to unicycle before. While a lot of the students struggle with the demanding task of learning to unicycle, a few of them seem to have easier to learn. One of those who quickly “get it” is Zack, who learns to unicycle at a quite astonishing pace. Through talking to Zack and through listening to him talking to other students, we realise that he has volunteered to take part in the project because he aspires to add unicycling ability to a range of other “acrobatic” capabilities, such as wakeboarding, trampoline jumping and walking on a slack line. Thus, he seems not to be focused primarily on the learning process, like many other students, but on *what he will be able to do* later on. Moreover, through his previous experience of practising wakeboarding and similar movements, he has developed a disposition to explore kinescapes on his own, without guidance from an instructor or coach. Unlike some other students, it does not bother him that neither his lecturer nor the researchers aspire to instruct him how to unicycle. Zack indicates that he experiments with different preliminary concepts of unicycling. Initially, he attempts to find the balance on the unicycle while stationary, but he soon realises that it is easier to keep the balance if he moves forward. In starting to focus on forward propulsion, he also realises that he has to “let go of his legs,” meaning that rather than “steer” the pedalling with his thinking, he focuses on where he is going or what will happen during the transportation (riding slalom between cones, for example). Put simply, this approach allows his “organism” to solve the movement problem. This does not mean, however, that he is entirely unaware of how the movement problem is actually solved while his focus is on what he wants to achieve. Rather, it means that solving the movement problem is part of his subsidiary awareness. If something unexpected happens, Zack is swiftly able to shift his focal attention in ways that enable him to solve any movement problems that arise on his way forward.

It seemed to us that Zack's practising was clearly affected not only by an aspiration to learn (how) to unicycle, but by an aspiration to be able to do spectacular tricks on a unicycle. Furthermore, unlike many students, he had few doubts that he would succeed in his venture to learn to unicycle. In fact, his “motoric” identity and self-confidence was frequently acknowledged and boosted by his peers. Zack seemed to be aware of (arguably from previous practise) that he needed to start from a preliminary concept of what unicycling ability means. He also seemed to realise that he needed to be able to swiftly abandon or re-configure the initial concept. His shift from stationary balancing to forward propulsion is a good example of this. In comparison to most other students, Zack moved forward at a significantly faster pace, which indicates that parallel to his ability to change or re-configure his tentative unicycling concept, he also managed to shift his focus of attention (percepts) in ways that allowed him to embody gradually more critical aspects which were necessary to develop his unicycling ability. Evidently, and probably due to his previous experiences of board sports culture (cf. Bäckström, 2014), Zack was “fluent” in kinesiocultural exploration. And so were other students, but not all. There were situations in the unicycling unit where students did not experience any learning, and where some of them decided to cease participation before the unit ended. One of these students was Josh.

In an exchange between Josh, another student teacher, Phil, and one of the researchers, Josh explains that he believes that unicycle ability is mainly about what he calls “core stability.” “If you keep track of your core stability, you can basically do anything. And if you can correct it with, like, swaying and stuff, then it will not be difficult, I think. Control of torso strength, everything else is secondary, I think.” In response to this, Phil wonders if Josh does not think that balance matters. “Balance—it all boils down to core stability,” answers Josh. In his logbook, Josh writes before practising unicycling the first session that it will be “Very exciting and I think it will be easy to find the rhythm!” When asked about what five words he associates with unicycling, Josh writes: “Coordination, balance, strength, torso (or core; our note) and fun.” When starting to practise, however, it is immediately clear to Josh that learning to unicycle was more challenging than he had anticipated. In his logbook he writes: “At first I thought it was about core stability. My thought was that as long as the core stability is there, then you can do anything! Unfortunately, very quickly, I realised that was the least I needed to think about.” The video recordings from the GoPro-cameras frequently show Josh struggling, either on his own or with the support of a friend, to mount the unicycle, find a minimal level of balance with support, and then attempting to pedal away from the support. He aborts most of his attempts almost before he starts them. Nevertheless, these attempts brought with them what could be described as some hope, at least judging by Josh's logbook entries:

I realised that if I hold my partner, it's much easier. My aha-experience was that if I'm not that tense, I can focus more on forward motion. After having been practising for a while, I realised that I had better balance if I stopped thinking too much on balancing!

Despite these temporarily experienced achievements, where Josh learned, for example, that it could be worthwhile to not think too much about certain things, i.e., to “let happen,” he concludes his first practise session like this: “it was difficult, and I totally lost my motivation. I had a picture that it would be a lot more fun than it actually turned out to be.” Josh kept practising for a couple of sessions, but without much success. During the third session, the GoPro-camera catches Josh talking to a fellow student about his experiences: “It's [i.e., unicycling] awful… it's not for me… I feel like a teenager, you know, when something's not fun and you give up, like.” Josh participates also on session four, but without much commitment. On the last day, Josh does not show up at all.

Like all students, Josh volunteered to participate in the unicycle unit. He probably looked forward to learning something new, at least this is what his logbook entries suggest (affect). He had a kind of initial concept about what unicycling would be about (i.e., core stability), but soon discovered that core stability was only a very minor aspect of unicycling ability. Unlike other students, who could offer quite a few metaphors for unicycling (which they also realised were not always entirely apposite: balancing on a slack line, bicycling, inline skating, for example), Josh struggled to come up with an alternative concept of unicycling, even though he realised that core stability was not “it.” Perhaps core stability was too far from a concept that would have helped Josh get moving. Josh never discovered any virtual domain of difference in itself. Instead, unicycling seemed to remain an amorphous chaos rather than a collection of structured problems.

Zack's experience was very different to Josh's. Zack seemed to discover a virtual domain of difference in itself during his practise. To him, unicycling was not an amorphous chaos, but a collection of structured problems. Since Josh could not come up with an alternative to his initial concept about core stability, he struggled to decipher the unicycling hieroglyphs. Or in other words, he did not find any aspect on which to focus his attention once he mounted the unicycle. Possibly, enforced by his initial idea that learning to unicycle would be fun, after a while, he “decided” that unicycling was not for him and left the practise. It should be noted that while Josh never admonished the researchers or the teacher educator who led the activities, neither researchers nor teacher educator offered him instruction beyond some suggestions on what he could try. Josh had plenty of experience of previously learning new movements, but unlike Zack, it seemed as if Josh was not assisted by these experiences in his exploration of the unicycling kinescape.

### Dancing

As discussed above, we selected dancing as one of the movement practises in the study because its norms, at least among most students and student teachers, differ from mainstream sport. This does not mean, that dancing is free of norms regarding “good or bad” or “who is supposed to do what,” in terms of, for example gender (e.g., Gard, 2003). Nor is the objective of dancing, in contrast to juggling and unicycling, that obvious to participants. The sort of dancing that we included in the project can be described as a short, choreographed routine to music. The students were invited, first, to explore a number of dance moves, such as chassé, grapevine and shuffle, but also what a “wave” could mean. The dancing took place to various kinds of music (three-stroke, and four-stroke in varying pulse and character) which were introduced by a teacher educator and which the students could explore further based on questions and video clips from YouTube. Then the students were put together in groups of four to five with the task of composing a 2–3-min routine which included some of the previously mentioned moves.

In a previous article (Nyberg et al., 2021), we offered an account of one student's—Robert—“learning journey” based on Ryle's and Polanyi's theoretical framework. This framework focuses to quite an extent on how participants approach challenging learning tasks, and specifically what they pay attention to in the kinescape. We believe that this is close to what Deleuze calls percepts, and to some extent also concepts. Robert typically approached the dance moves, all of which were new to him, through a number of strategies. These strategies consisted of “occupying the vantage point,” “following” and “investigating one chosen path.”

*Occupying the vantage point*: “When each new dance move is introduced, either by the teacher or through a video, Robert takes a position as an observer. As observer, he does not move except for lightly stroking or pulling his beard. He just watches” (Nyberg et al., 2021, p. 286).

*Following*: “Robert imitates and follows either actively with the sharp awareness of his way of moving, or passively in terms of following on a leash, through a landscape without knowing where he is heading. In this latter kind of action, he is merely trying to catch up and become more aware of the leaders' ways of moving than his own” (p. 287).

*Investigating one chosen path*: “At times, Robert seems to analyse his way of moving by dividing the movement up into parts […] and then putting them together. […] Robert does, however, also attempt to see the “whole area” by trying the dance move without breaking it into parts” (p. 287).

In the previous article, our analysis was based on video recordings. By adding information from Robert's logbook, we can provide more information about what the movements meant to him (concepts), what he was paying attention to (percepts), and, importantly, what affected his learning process. After the initial “trials” during the first practise session, he wrote:

Without music: difficult to coordinate. Feel stiff in the feet, knee and hip joints […]. Difficult movements despite the freedom to be creative due to no relationship with dance. With music: easier to find a flow in the movements. Depending on the music, you can make the movements softer and harder. The first time, you're not completely comfortable with the task, neither with nor without music (Robert's logbook, first session).

Same movements as the first session. Is going better now, probably because you recognise the movements. Is now with people in the group you're more comfortable with than last time, plus that one of us is more beginner than I am. Kick ball change is a movement I have previous experience of since I'm a former football player and have now been coaching for 4 years and teach shots basically every week all year round. The dancing was more fun today than the first session, probably due to the fact that you (I) nailed the movements better than the first time (Robert's logbook, second session).

Information from the logbook indicates how Robert feels that his unfamiliarity with the dance moves makes him uncertain and tense. He struggles with making sense of a lot of the movements (concept) and does not know what to focus on (percept)—which is expressed in the fact that he often stands still, silently observing, pulling his beard (affect). Sometimes, he can make sense of the step because it reminds him of something else. The kick ball change step, for instance, resembles a football shot (concept) for Robert. The recognition gets him moving (affect), and it also helps him focus on key aspects of the movement that makes him experience improvement and which encourages him to explore further. We noticed this improvement in our video recordings:

Robert imitates the instructor on the video and he manages this step (kick ball change) immediately. However, when trying further he leaves out the end of “the change.” But he continues to imitate. He gets it again and soon he also moves his arms. It seems that he is confident in doing the kick ball change! (Nyberg et al., 2021, p. 287).

In the subsequent group work, Robert kept a rather low profile. His focus was mainly on following another student, who took the lead in the group work with composing and performing the routine. In his logbook, Robert writes: “The show went well. Comfortable in the group. There were some difficulties left in the steps but I myself set the movements perfectly okay.”

## Discussion

Above, we have illustrated how Deleuzian thinking concerning an apprenticeship in signs based on a pedagogy of the concept can be used to explore genuine movement learning practise, which was one purpose of this article. We will now discuss the empirical examples *with* Deleuzian thinking. Subsequently, we will discuss the second part of the purpose, which was how this approach can stimulate thinking and understanding of genuine movement learning and provide insights about pedagogical implications of this perspective in movement educational settings.

In our empirical examples, we have offered accounts of what we understand as genuine learning. Adele and Beth, Zack, and Robert, all in slightly different ways, managed to learn to respectively juggle, unicycle and dance following an initial disorientation (cf. Bogue, 2004). As a result of this disorientation, they managed to move beyond their ordinary operations (cf. Semetsky, 2007); they passed through interpretative illusions until they grasped difference in itself in its immanent differentiation of the actual, to paraphrase Bogue (2004, p. 341). Or put simply, they learned to juggle, unicycle or dance, at least in a fashion, given what is socioculturally considered as dancing, unicycling or juggling ability in the particular context. The notion of an apprenticeship in signs signals in a movement learning context that learning is about deciphering what it means to move in particular ways, and if I want to move in certain ways, or for certain purposes, I need to pay attention to what my way of moving *does* as I move. This is apparent in all the empirical examples. For example, it was key for Adele and Beth in attempting to juggle that they managed to discern the difference between juggling in a circular pattern and juggling in a cascade pattern and what this meant for their possibility to further develop their juggling capabilities. Similarly, it was crucial that Zack managed to discern the difference between forward propulsion and stationary balancing on the unicycle for his possibility to further develop his unicycling abilities; and it was crucial for Robert to be able to compare certain dance moves to other movements that he was more familiar with for his possibility to further develop his dancing abilities.

In fact, we believe that our previous use of the concept of “mind” (Ryle, 2009) and “personal knowledge” (Polanyi, 2012) could have helped us draw similar conclusions. Turning do Deleuze adds to the analysis how perception, in all three empirical examples, was sharpened and sometimes also relocated parallel to the development of new/altered concepts. Relocating perception allowed for new aspects—new signs—to appear before the learners' attention; aspects that were significant for the learners' possibilities to further develop their movement capabilities. Finally, new/altered concepts and changed percepts also meant that Adele, Beth, Zack and Robert, unlike Josh, found inspiration to continue exploring their respective kinescapes, not least because the discovery was experienced as increased opportunities for action. While Josh indeed attempted to learn to unicycle, for various reasons he seemed not to get past experiencing the practise as an amorphous chaos. Josh and some other students never managed to decipher the signs that the practise emitted in ways that they reached a potential actualisation of the problem that was recognisable as “ability” in the particular context. It is difficult indeed to tell why some learned while others did not, however, we believe that an apprenticeship in signs based on a pedagogy of the concept still offers some pedagogical reflections concerning how teaching can be approached.

Obviously, a Deleuzian approach to teaching hardly relies on direct instruction in the manner of “Do as I tell you,” as is often the case in linear approaches (cf. Chen et al., 2016; Bedard et al., 2020). But neither is it, according to Deleuze, useful to expect that people will learn simply when someone shows the right execution of the movement in the manner of “Do as I do.” Rather, Deleuze (1994, p. 23) proposes that: “Our only teachers are those who tell us to “do with me,” and are able to emit signs to be developed in heterogeneity rather than propose gestures for us to reproduce.” So, a teacher or coach may show, but not expect the learner to learn directly from the example. Rather, teaching can, like learning, also be regarded as an exploration. Or as Bogue (2004, p. 341) suggests:

One cannot teach the truly new in its newness, but one can attempt to induce an encounter with the new by emitting signs, by creating problematic objects, experiences or concepts. Hence, the pedagogy of signs entails first a critique of codes and conventions, an undoing of orthodox connections, and then a reconnection of elements such that the gaps between them generate problems, fields of differential relations and singular points. Such teaching, however, is itself a form of learning, for it proceeds via an encounter with signs and an engagement with problems. To teach is to learn, finally, since for Deleuze genuine teaching and learning are simply names for genuine thought. The goal of teaching and learning is to think otherwise, to engage the force of that which is other, different and new. What Deleuze details in his accounts of learning and teaching is that dimension of education that inspires all true students and teachers—the dimension of discovery and creation within the ever-unfolding domain of the new. It is also the dimension of freedom, in which thought escapes its preconceptions and explores new possibilities for life.

Teaching and learning new movements, then, can be seen as a collaborative exploration between teacher and learner of the signs that participation in a movement practise emits. The aspiration of a teacher may well be to teach a learner a particular way of moving, but the teacher can never take for granted how a learner understands and approaches the learning of this movement. Moreover, since learning is open-ended (Aggerholm, 2021), there is no use for a teacher to expect that a learner should master a movement or movement practise in any “final” way. “Getting it right” is not about narrowing down how a movement should be executed and experienced. On the contrary, “getting it right” can be understood as developing an ever more complex way of experiencing a movement, which will help the learner to adapt, change and develop the movement in a variety of ways in a multitude of new situations. The task of a movement teacher could, then, be to accompany learners in their kinesiocultural exploration of kinescapes, possibly as “critical friends” (Costa and Kallick, 1993).

An apprenticeship in signs encourages in our interpretation that teachers, coaches and instructors offer tasks that require, or encourage, learners to ask questions about what it means (or how it feels) to move in certain ways. Asking questions about what it means/how it feels to move in certain ways is, however, not tantamount to pilotage in the sense that the questions are directly intended to have learners move in the intended ways, but to help learners sharpen their senses and, like unicycle equilibrist Zack, develop a strategy where the learner “works” with various concepts of what it means/how it feels to move in certain ways. Such a strategy involves also the ability to be perceptive to “what happens” and to swiftly shift the focus of attention in order to solve impending movement problems. The attentive reader will note here that we integrate a pedagogy of the concept into the apprenticeship in signs, which involves questions about “what do you pay attention to?” (percepts) and “what could it mean?” (concepts). What is left out so far is affects, that is, “what gets things moving?” (affects). One challenging pedagogical question is how to involve people in practise in the first place. While we can shed limited information on this issue, it is clearer from our study that progress in learning is one important aspect. Pedagogically, this means that teachers, coaches and instructors need to continuously consider the progress, or lack of progress, that the learners experience in their practise. Included in this pedagogical approach may also be to help learners becoming aware of their progress (or lack of progress).

Thinking the triadic relation between concepts, percepts and affects means that what is becoming perceived, the meaning of what is perceived and the drive to continue practising is interwoven. Coming to know what structural equation modelling means, what it could be useful for, also means that you perceive it differently, which makes you want to explore—or use—it in various situations. The same goes for movements. Getting to know the juggling cascade pattern, how to balance on a unicycle or the kick ball change dance move also means that you perceive it differently and want to explore how it could be used to juggle, unicycle and dance. Consequently, the pedagogical strategy of a teacher, coach or instructor could be to become attentive to what movements mean to learners, both as parts of the practise (e.g., techniques, steps, etc.) and the whole practise, and how they are perceived. Additionally, teachers, coaches and instructors could offer tasks or ask questions that will encourage learners to explore movements—or kinescapes—parallel to reflecting upon what is required in terms of participation in order to be able to participate purposefully.

This open-ended approach to teaching and learning offers, we believe, a useful approach to interrogate also the effects of embedded power structures. According to previous research, sociocultural norms relating to for example, class (Evans, 2004), disability (Fitzgerald, 2008), and gender (Gard, 2003) characterise movements and movement practises in the sense that learners prefer to move, and to learn moving, in certain ways, and that they perceive and understand movements differently. Kinesiocultural exploration may involve paying attention to, negotiating, and playing with, these norms in a manner that allows learners to go beyond stereotypical movement aspirations, ways of apprehending movements and ways of moving. It could be worthwhile to play with these norms because it “stretches” the learners' movement experiences. It helps learners to develop more complex ways of doing and experiencing movements. In Bogue's (Bogue, 2004) words, this is about an undoing of orthodox connections; a way of escaping preconceptions about movements and an exploration of new possibilities for moving!

Relating the Deleuzian-inspired approach to current approaches to movement learning, we feel that there is great affinity between non-linear approaches and the here presented approach (e.g., Renshaw et al., 2010; Davids et al., 2012; Chow et al., 2015). However, to some extent, we still perceive that non-linear approaches, such as the constraints-led approach (Renshaw et al., 2016; Renshaw and Chow, 2019), is working with assumptions about that movements are “programmed,” albeit not in a linear fashion, and that the end goal of the practise is that the learner should be able to move in particular ways. Moreover, the sociocultural norms, including how these materialise as equipment, localities with a certain design, and more, are rarely brought into the equation. Gender or other social norms are rarely considered as constraints and affordances (for an exception, see Larsson et al., 2021).

In *A Thousand Plateaus*, Deleuze and Guattari (2013) asserts that “[i]f the three ages of the concept are the encyclopaedia, pedagogy, and commercial professional training, only the second can safeguard us from falling from the heights of the first into the disaster of the third” (p. 12). We take it that this means that, for Deleuze and Guattari, there is indeed room for teaching/a teacher. However, “the teacher” is neither a well-informed lecturer nor an expert demonstrator, but a fellow explorer; in the case of genuine movement education, this means a fellow kinesiocultural explorer of kinescapes.

## Data Availability Statement

The raw data supporting the conclusions of this article will be made available by the authors, without undue reservation.

## Ethics Statement

The studies involving human participants were reviewed and approved by Regional Research Ethical Review Committee (Gothenburg). Written informed consent from the participants' legal guardian/next of kin was not required to participate in this study in accordance with the national legislation and the institutional requirements.

## Author Contributions

HL is the first author of the article. HL drafted the text and then HL, GN, and DB have worked together with the manuscript and approved the submitted version. The article draws on empirical material from the MOVE project, with DB as project manager. The work in the project has been evenly distributed between DB, GN, and HL with respect to design, data production, and analysis.

## Funding

The MOVE project was funded by The Swedish Research Council, grant no. 2017-03471.

## Conflict of Interest

The authors declare that the research was conducted in the absence of any commercial or financial relationships that could be construed as a potential conflict of interest.

## Publisher's Note

All claims expressed in this article are solely those of the authors and do not necessarily represent those of their affiliated organizations, or those of the publisher, the editors and the reviewers. Any product that may be evaluated in this article, or claim that may be made by its manufacturer, is not guaranteed or endorsed by the publisher.
